# Supplementary material for: Association between oral microbiome and seven types of cancers in East Asian population: a two-sample Mendelian randomization analysis
Source: Front Mol Biosci. 2023 Nov 21;10:1327893. doi: 10.3389/fmolb.2023.1327893 (PMC10702768; doi:10.3389/fmolb.2023.1327893)
Supplement: Supplementary file 1 [file DataSheet1.ZIP › Supplementary Materials/MR plots of saliva/saliva═╝/Breast cancer/pheno.1026_to_breast cancer_leave_one_out.pdf]

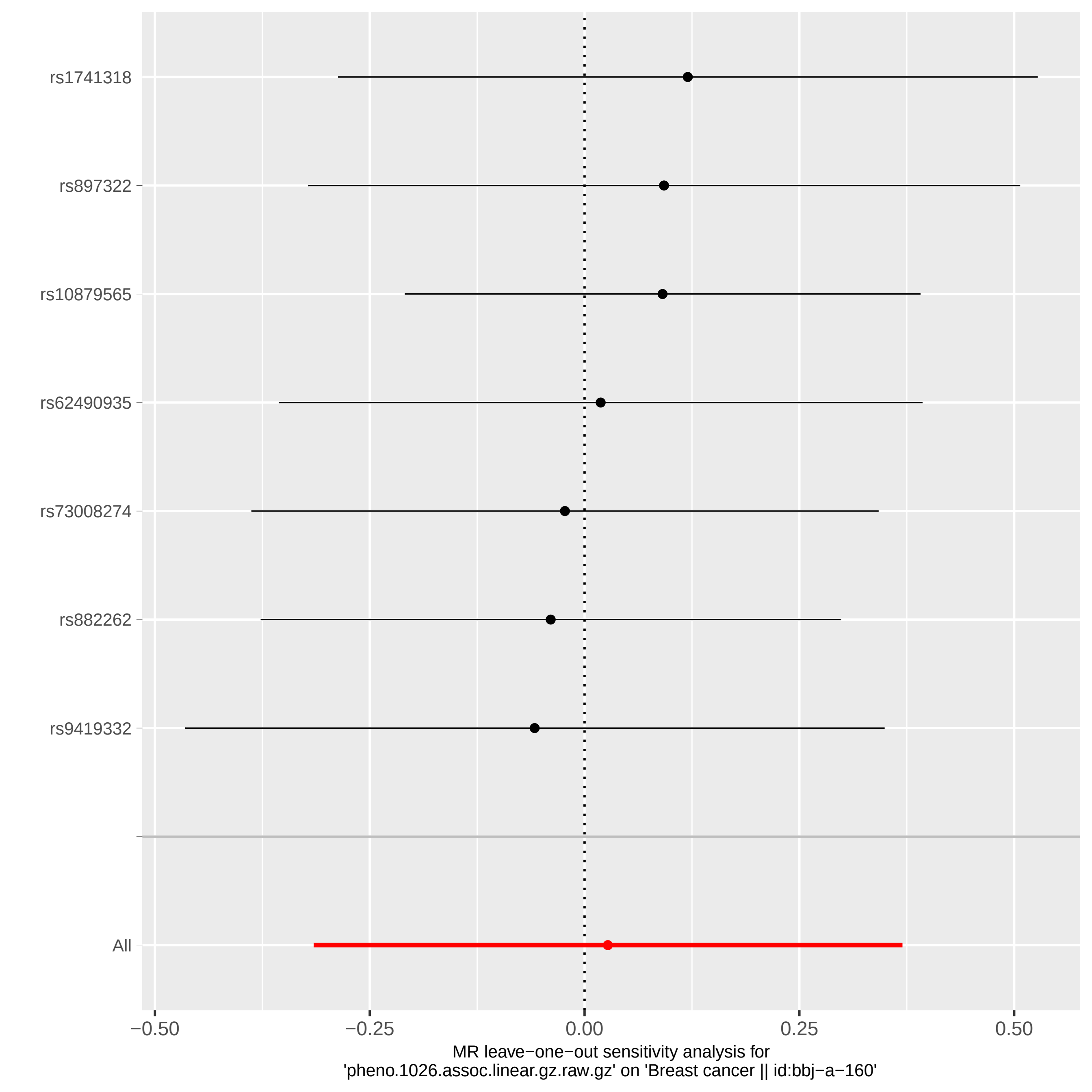
rs1741318
rs897322
rs10879565
rs62490935
rs73008274
rs882262
rs9419332
All
−0.50
−0.25
0.00
0.25
0.50
MR leave−one−out sensitivity analysis for
'pheno.1026.assoc.linear.gz.raw.gz' on 'Breast cancer || id:bbj−a−160'
